# The Class A β-Lactamase Produced by *Burkholderia* Species Compromises the Potency of Tebipenem against a Panel of Isolates from the United States

**DOI:** 10.3390/antibiotics11050674

**Published:** 2022-05-17

**Authors:** Scott A. Becka, Elise T. Zeiser, John J. LiPuma, Krisztina M. Papp-Wallace

**Affiliations:** 1 Research Service, Veterans Affairs Northeast Ohio Healthcare System, Cleveland, OH 44106, USA; scott.becka@va.gov (S.A.B.); elise.zeiser@va.gov (E.T.Z.); 2 Department of Pediatrics, University of Michigan Medical School, Ann Arbor, MI 48109, USA; jlipuma@med.umich.edu; 3 Departments of Medicine and Biochemistry, Case Western Reserve University, Cleveland, OH 44106, USA

**Keywords:** β-lactamase, *Burkholderia*, β-lactam, PenA, carbapenemase, tebipenem, carbapenem, AmpC

## Abstract

Tebipenem-pivoxil hydrobromide, an orally bioavailable carbapenem, is currently in clinical development for the treatment of extended-spectrum β-lactamase- and AmpC-producing Enterobacterales. Previously, tebipenem was found to possess antimicrobial activity against the biothreat pathogens, *Burkholderia pseudomallei* and *Burkholderia mallei*. Thus, herein, tebipenem was evaluated against a panel of 150 curated strains of *Burkholderia cepacia* complex (Bcc) and *Burkholderia gladioli*, pathogens that infect people who are immunocompromised or have cystic fibrosis. Using the provisional susceptibility breakpoint of 0.12 mg/L for tebipenem, 100% of the Bcc and *B*. *gladioli* tested as being provisionally resistant to tebipenem. Bcc and *B*. *gladioli* possess two inducible chromosomal β-lactamases, PenA and AmpC. Using purified PenA1 and AmpC1, model β-lactamases expressed in *Burkholderia multivorans* ATCC 17616, PenA1 was found to slowly hydrolyze tebipenem, while AmpC1 was inhibited by tebipenem with a *k*_2_/*K* value of 1.9 ± 0.1 × 10^3^ M^−1^s^−1^. In addition, tebipenem was found to be a weak inducer of *bla*_PenA1_ expression. The combination of the slow hydrolysis by PenA1 and weak induction of *bla*_PenA1_ likely compromises the potency of tebipenem against Bcc and *B*. *gladioli*.

## 1. Introduction

The discovery of tebipenem-pivoxil, a novel orally bioavailable carbapenem, was first reported in the 1990s by Wyeth Lederle Japan, Co., Ltd. in Japan and was approved for pediatric clinical use in Japan in 2009 [1,2,3]. At that time, tebipenem was found to be highly active against *Streptococcus pneumoniae* and β-lactamase-nonproducing ampicillin-resistant *Haemophilus influenzae*, two pathogens that were increasingly prevalent in Japan [2,4,5].

Due to the scourge of multi-drug-resistant gram-negative bacterial infections in the United States, tebipenem-pivoxil hydrobromide, a slightly modified formulation that improves drug properties (e.g., stability), is currently in clinical development by Spero Therapeutics in the US for the treatment of multi-drug-resistant gram-negative pathogens [6]. In addition to tebipenem’s antimicrobial activity against gram-positive pathogens, tebipenem has demonstrated a potency against strains of *Escherichia coli*, *Klebsiella pneumoniae*, *Enterobacter aerogenes* and *Proteus mirabilis,* producing extended-spectrum-β-lactamases (ESBLs) and plasmidic AmpC β-lactamases [7,8,9,10,11]. The inhibitory activity of tebipenem against Enterobacterales primarily lies in its ability to inactivate PBP2 [12]. Notably, the phase 3 clinical trial, ADAPT-PO, revealed that oral tebipenem was non-inferior to intravenous ertapenem for the treatment of complicated urinary tract infections and acute pyelonephritis [13].

In 2013, tebipenem was found to demonstrate some activity against the biothreat pathogen, *Burkholderia pseudomallei* [14]. Furthermore, in 2021 tebipenem was evaluated against a broader panel of biothreat pathogens, including *B*. *pseudomallei* and *Burkholderia mallei,* and minimum inhibitory concentrations (MICs) ranged between 1–4 mg/L and 0.25–1 mg/L, respectively [15]. The *Burkholderia* genus encompasses more than 30 species of mammalian pathogens. Major human and animal pathogens include *B. mallei*, *B. pseudomallei* complex, *Burkholderia cepacia* complex (Bcc), and *Burkholderia gladioli*. Populations particularly susceptible to acquiring *Burkholderia* spp. infections include those who are immunocompromised. For Bcc and *B*. *gladioli*, individuals with cystic fibrosis (CF) and chronic granulomatous disease are most vulnerable to infections [16,17,18,19,20]. Select β-lactam antibiotics (e.g., meropenem, ceftazidime) are often “first-line” agents and can be effective as a treatment option for infections due to Bcc and *B*. *gladioli*; unfortunately, their potency is declining [21,22,23,24]. *Burkholderia* spp. produce at least two chromosomal β-lactamases, a PenA-like family class A β-lactamase and an AmpC-like class C β-lactamase [25,26,27,28,29,30,31]. PenA1 and AmpC1 from *Burkholderia multivorans*, a member of the Bcc, were previously microbiologically and biochemically characterized, and PenA1 was found to be a carbapenemase with a very broad spectrum [32], while AmpC1 possessed a narrow spectrum that included some cephems [31]. In addition, some strains of Bcc also express an OXA class D β-lactamase [25,26,27,28,29,30,31]. In Bcc and *B*. *gladioli*, the expression of *bla*_PenA_ and *bla*_AmpC_ is regulated by PenR_A_, similar to the Enterobacterales’ *bla*_AmpC_/AmpR system [28,30,33]. Specifically, upon exposure to an inducing β-lactam, such as imipenem, the balance of the muropeptides within the bacteria is altered, which changes the repressor function of PenR_A_, and *bla*_PenA_ and *bla*_AmpC_ are expressed [30,31,33].

Previously, we have found that β-lactams and β-lactam-β-lactamase inhibitor combinations are some of the most potent antimicrobials against multi-drug- and extensively-drug-resistant Bcc and *B*. *gladioli* obtained from persons with CF in the US [23,34,35,36]. However, most of these agents are only available in intravenous formulations. Thus, a critical need exists to continue to explore novel treatment options, especially orally bioavailable agents, against Bcc and *B*. *gladioli*. Herein, the activity of tebipenem against Bcc and *B*. *gladioli* was explored, along with biochemical interactions of tebipenem with PenA1 and AmpC1, and the impact of tebipenem on the expression of *bla*_PenA1_ and *bla*_AmpC1_.

## 2. Results

### 2.1. Tebipenem Does Not Demonstrate Clinically Relevant Antimicrobial Activity against Bcc and B. gladioli

**Figure 1:**
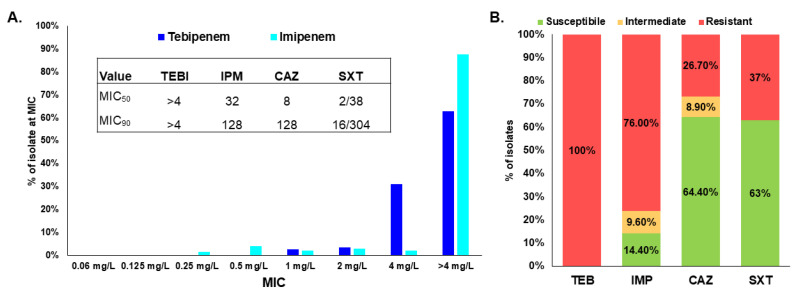
(**A**) Bar graph showing the % of isolates that had a specific MIC value. Inset: MIC_50_ and MIC_90_ values for antibiotics in next panel [34]. (**B**) Bar graph comparing the % susceptible, % intermediate, and % resistant of the isolates for tebipenem (TEB), imipenem (IMP), another carbapenem [23], and ceftazidime (CAZ) and trimethoprim-sulfamethoxazole (SXT), two first-line agents [23] used to treat infections caused by *Burkholderia* species.

*E. coli* ATCC 25922 and *P. aeruginosa* ATCC 27853 were used as controls for tebipenem integrity; the controls tested within the anticipated quality control ranges (Table 1). Thus, agar dilution testing was confirmed to provide equivalent results to the reference broth microdilution, as the agar dilution MICs for the quality control strains were within the proposed quality control ranges for tebipenem [37]. Antimicrobial susceptibility testing using the agar dilution methodology was further used to determine the MICs for a well-characterized panel of 150 clinical isolates of Bcc and *B*. *gladioli* [23,30,31,34,35,36,38]. One hundred percent of the isolates tested provisionally resistant to tebipenem, with all isolates possessing an MIC ≥ 1 mg/L (Figure 1A and Supplementary Table S1). The majority of the strains tested (63%) produced an MIC > 4 mg/L. A limitation of this study was that endpoint MICs were not determined; this decision was based on the provisional breakpoints for tebipenem. Different patterns in the susceptibility profiles to tebipenem based on *Burkholderia* species were not observed. The tebipenem MICs were compared to those of another carbapenem, imipenem; however, tebipenem MICs were overall lower when compared to imipenem (Figure 1A). However, using the percent provisional susceptibility to tebipenem and imipenem susceptibility breakpoint of *Pseudomonas aeruginosa* for *Burkholderia* species (as an imipenem breakpoint is not available), 76% of the isolates tested resistant to imipenem (Figure 1B) [23]. Comparatively, strains were 64.4% and 63% susceptible to the first-line agents ceftazidime and trimethoprim-sulfamethoxazole, respectively, used to treat infections due to Bcc and *B*. *gladioli*; in general, these are a highly resistant subset of bacteria [23]. Moreover, compared to tebipenem, MIC_90_ values for these former agents were in the resistant range (Figure 1, inset) [34,37].

### 2.2. Tebipenem Is Slowly Hydrolyzed by PenA1 but Inhibits AmpC1, but Eventually Forms a Stable Complex with Both β-lactamases

**Figure 2:**
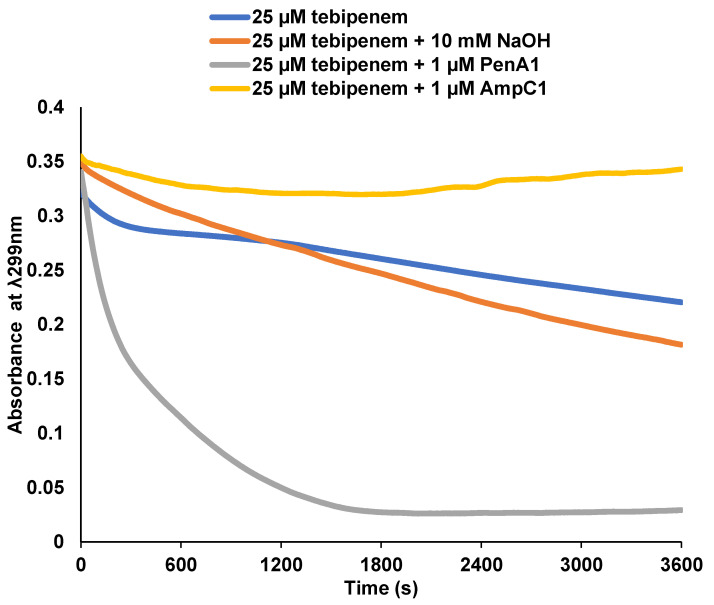
Progress curves showing the breakdown of 25 µM tebipenem over time alone (blue), and hydrolysis by the addition of 10 mM sodium hydroxide (NaOH) (orange), 1 µM PenA1 (gray) or 1 µM AmpC1 (yellow).

**Figure 3:**
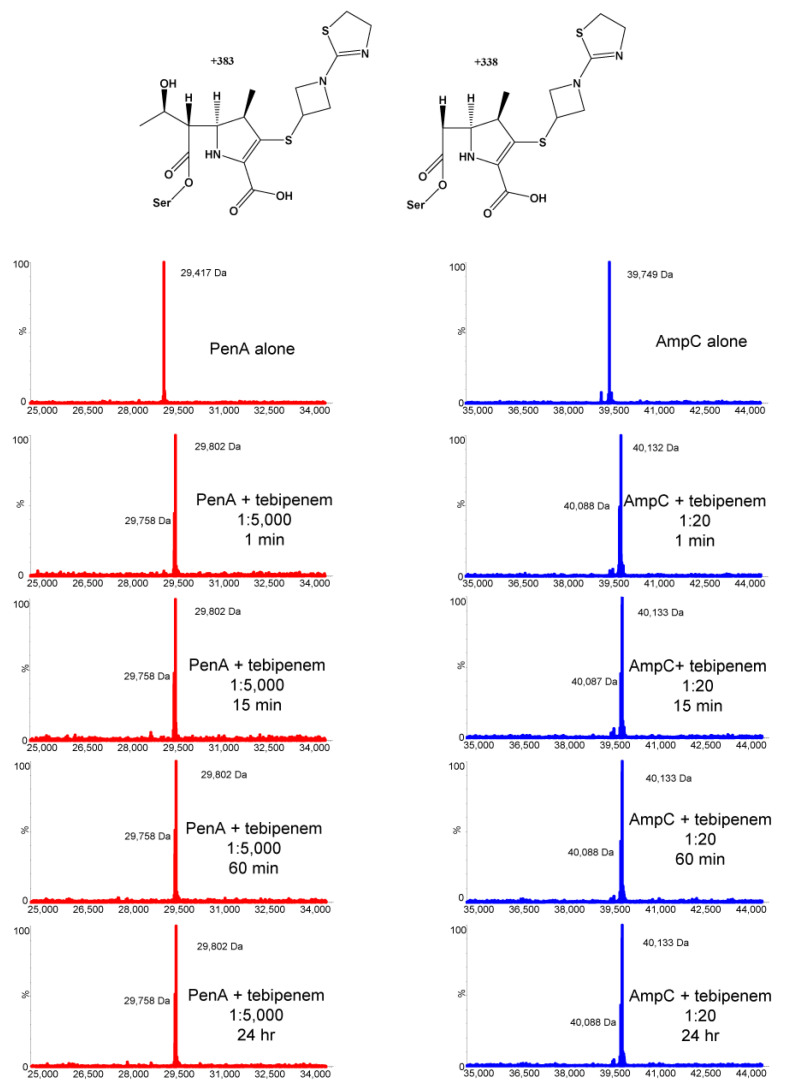
ESI-MS of PenA1 (red) and AmpC1 (blue) with tebipenem at various time points (1 min, 15 min, 60 min, and 24 h) to reveal the intermediates formed upon incubation, i.e., acyl-enzyme (top left) and acyl-enzyme with loss of R1 hydroxyethyl side chain (top right). Both enzymes are also depicted without tebipenem to show the apo-enzyme’s molecular weight. The ratio of enzyme to tebipenem was chosen based on the *t*_n_ at 15 min; see Table 2.

The kinetic characterization of purified PenA1 and AmpC1 from *B*. *multivorans* ATCC 17616 revealed that PenA1 slowly hydrolyzed tebipenem (Figure 2). One µM PenA1 hydrolyzed 25 µM tebipenem within 30 min. Conversely, AmpC1 did not hydrolyze tebipenem and lowered the background hydrolysis of tebipenem, likely due to AmpC1 being in an acyl-enzyme complex with tebipenem. The *K*_i app_ of PenA1 for tebipenem was 4.7 ± 0.5 µM; however, 4000 molecules of tebipenem were turned over in 15 min by PenA1 before it was inactivated, thus resulting in non-determinable *k*_2_/*K* and *k*_off_ values (Table 2). As AmpC1 did not hydrolyze tebipenem, *K*_i app_, *k*_2_/*K*, and *k*_off_ values were determinable at 22 ± 2 µM, 1.9 ± 0.1 × 10^3^ M^−1^s^−1^, and 3 ± 1 × 10^−4^ s^−1^, respectively (Table 2). In addition, the number of molecules of tebipenem hydrolyzed in 15 min (*t*_n_) before AmpC was inactivated was only 10. This is 400-fold less than that observed with PenA1. To assess the stability of the PenA1-tebipenem and AmpC1-tebipenem complexes over time and to determine if tebipenem is modified over time, ESI-MS was employed using enzyme-to-inhibitor (E:I) ratios greater than the *t*_n_ at 15 min for each enzyme. At 1:5000 and 1:20 E:I ratios for PenA1 and AmpC1, respectively, tebipenem remained bound up to the last time point of 24 h (Figure 3). Thus, once the tebipenem acylated PenA1 and AmpC1, it remained in a stable complex. In addition, ESI-MS revealed that the hydroxyethyl side chain of tebipenem was lost upon acylating both PenA1 and AmpC1 with a loss of 45 Da. The loss of the hydroxyethyl side chain of carbapenems has previously been described by others [39,40,41,42,43].

### 2.3. Tebipenem Is a Minor Inducer of bla_PenA1_ Expression

**Figure 4:**
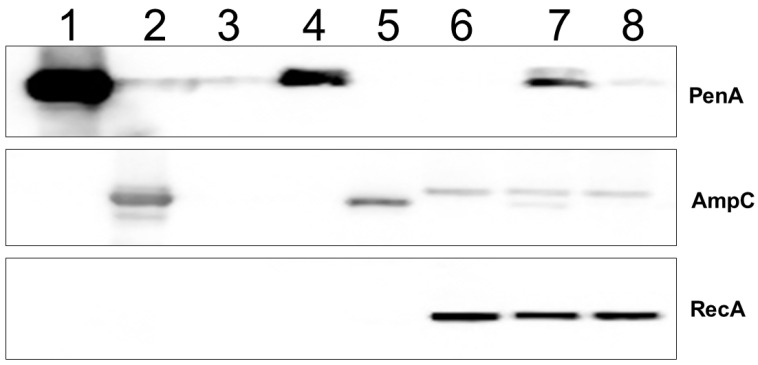
Immunoblotting using anti-PenA1 peptide and anti-AmpC1 polyclonal antibodies as well as an anti-RecA antibody, as an internal standard for the uninduced and induced *B*. *multivorans* samples. *B*. *multivorans* ATCC 17616 was grown to log phase (OD_600 nm_ ~0.6) in LB, and then cultures were either maintained in LB only (lane 6), or 1 mg/L of imipenem, a known inducer of *bla*_PenA1_ and *bla*_AmpC1_ expression [30], was added (lane 7), or 1 mg/L tebipenem was added (lane 8), and cultures were grown for an additional hour, pelleted, and crude extracts were prepared for immunoblotting. Purified PenA1 (lane 1), purified AmpC (lane 2), *E*. *coli* DH10B with pBC SK(+) (lane 3), *E*. *coli* DH10B with pBC SK(+) *bla*_PenA1_ (lane 4), and *E*. *coli* DH10B with pBC SK(+) *bla*_AmpC1_ (lane 5) were used as controls.

Using Western blotting against PenA1 and AmpC1, tebipenem was found to weakly induce the expression of *bla*_PenA1_, as evidenced by the faint band in the lane analyzing *B*. *multivorans* ATCC 17616 treated with 1 mg/L of tebipenem (Figure 4, lane 8). Conversely, a basal level of AmpC1 was detected in all lanes carrying crude extracts of *B*. *multivorans* ATCC 17616 (Figure 4, lane 7). Moreover, AmpC without its signal peptide was observed only in the plus imipenem lane, likely due to the samples being extracted prior to AmpC reaching the periplasm where the signal peptide is removed.

## 3. Discussion

Based on the provisional breakpoints provided by the sponsor of this study [15], tebipenem was found to lack antimicrobial activity against a strain panel of difficult-to-treat clinical isolates of Bcc and *B*. *gladioli*. The lack of activity is likely due to the slow hydrolysis of tebipenem by PenA1 as well as the weak induction of *bla*_PenA1_ expression. Thus, tebipenem is a slow substrate for PenA1; conversely, tebipenem inactivates AmpC1. These data are reminiscent of those previously observed with imipenem [23,32]. Imipenem is also slowly hydrolyzed by PenA, but not AmpC1 [32], and 76% of the isolates tested herein are resistant to imipenem using the Clinical Laboratory and Standards Institute’s (CLSI’s) *P. aeruginosa* susceptibility breakpoint of ≤2 mg/L [23,37]. The addition of a PenA1 inhibitor would likely improve the potency of tebipenem against Bcc and *B*. *gladioli*; however, as tebipenem-pivoxil hydrobromide is not being partnered with a β-lactamase inhibitor, this was not tested in this study. Indeed, adding relebactam to imipenem improved imipenem’s potency against this panel of isolates with 71.4% testing susceptible to the imipenem-relebactam combination [38]. Importantly, when tebipenem was tested against *B*. *pseudomallei* and *B. mallei*, MIC_90_ values of 2 mg/L and 1 mg/L, respectively, were observed [15]. Thus, tebipenem was more active versus that strain panel compared to the one tested herein; the MIC_90_ value was >4 mg/L (Figure 1, inset). The *B*. *pseudomallei* and *B. mallei* MICs ranged between 1–4 mg/L and 0.25–1 mg/L, respectively; based on those datasets and the provisional susceptibility breakpoint for tebipenem, these pathogens would also be provisionally resistant to tebipenem [15]. Given the provisional breakpoints for tebipenem, tebipenem lacks sufficient antimicrobial activity against Burkholderial pathogens.

## 4. Materials and Methods

*Susceptibility Testing.* An extensively characterized panel of 150 MDR clinical strains [44], including 140 Bcc (*B. ambifaria*, *B. arboris*, *B. cenocepacia*, *B. cepacia*, *B. contaminans*, *B. diffusa*, *B. dolosa*, *B. multivorans*, *B. pseudomultivorans*, *B. pyrrocinia*, *B. seminalis*, *B. stabilis*, *B. ubonensis* and *B. vietnamiensis*) and 10 *B. gladioli* obtained from the Burkholderia cepacia Research Laboratory and Repository (University of Michigan) were phenotypically characterized using agar dilution MICs (tebipenem range: 0.06–4 mg/L) by using a Steers replicator that deposited 10^4^ colony forming units of bacteria per spot. All Burkholderia species were isolated and speciated as previously described [23]. In addition, *E. coli* ATCC 25922, *P. aeruginosa* ATCC 27853, and *B. multivorans* ATCC 17616 were used as control strains. Provisional MIC interpretations for tebipenem were provided by Spero Therapeutics: susceptible ≤ 0.12 mg/L, intermediate 0.25 mg/L, and resistant ≥0.5 mg/L [15]. Provisional breakpoints are used when official breakpoints have not been assigned by the Clinical Laboratory Standards Institute, European Committee on Antimicrobial Susceptibility Testing, or another leading agency that publishes antibiotic breakpoints for microorganisms.

*Protein Purification and Steady-State Kinetic Analysis of Inhibition.* The purification protocols for PenA1 and AmpC1 were previously described [31,32]. Using base hydrolysis (i.e., sodium hydroxide (NaOH)), the absorbance wavelength and extinction coefficient (–12,090.76 M^−1^cm^−1^ at 299 nm) for tebipenem was determined using the Beer–Lambert law. The stability of tebipenem for each β-lactamase was assessed by incubating 25 µM tebipenem with 1.0 µM of purified PenA1 or AmpC1—10 mM NaOH was used as a control base—and hydrolysis curves were monitored at ~25 °C for 1 h using an Agilent 8453 Diode Array spectrophotometer for measurements in 10 mM phosphate-buffered saline, pH 7.4 (PBS). In addition, *K*_i app_, the *k*_2_/*K* value or acylation rate, *k*_off_ or the off-rate and partition ratios (*t*_n_) at 15 min were attempted and determined if feasible, using previously described methods [45,46].

*Timed-Electrospray Ionization-Mass Spectrometry (ESI-MS).* ESI-MS was performed to assess the timing of the reaction course as well as discern the nature of the intermediates formed upon the reaction of tebipenem with the β-lactamases. The purified PenA1 and AmpC1 β-lactamases were incubated with tebipenem at 1:5000 and 1:20 enzyme-to-inhibitor (E:I) ratios for 1 min, 15 min, 60 min, and 24 h. A Waters SYNAPT G2-Si quadrupole-time-of-flight mass spectrometer equipped with a Waters Acquity H class Ultra Performance Liquid Chromatography (UPLC) was used according to previously described methods [47].

*Induction Assays and Western Blotting.* To measure β-lactamase induction in *B. multivorans*, Western blots were used to assess the level of PenA1 and AmpC1 induction; the methodology was previously described [30,31]. Briefly, polyclonal anti-PenA peptide, anti-AmpC, and anti-RecA antibodies were previously generated in rabbits for use against *B*. *multivorans* [30,31]. An anti-RecA antibody was used as a loading control for cellular samples. Strains were grown in lysogeny broth (LB) to an optical density at 600 nm (OD_600 nm_) of ~0.6, after which sub-MIC concentrations—that do not alter the growth of the bacteria—of imipenem (1 µg/mL), a known inducer [30], and tebipenem (1 µg/mL) were added. The cells were grown for an additional hour, and samples were prepared for Western blotting, as previously described [30,31]. Purified PenA1 and purified AmpC as well as *E*. *coli* DH10B with pBC SK(+), *E*. *coli* DH10B with pBC SK(+) *bla*_PenA1_, and *E*. *coli* DH10B with pBC SK(+) *bla*_AmpC1_ grown in LB only were used as controls.

## Figures and Tables

**Table 1:** MICs values in mg/L for selected control strains.

Control Strain	Tebipenem
*B. multivorans* ATCC 17616	>4
*E. coli* ATCC 25922	≤0.06
*P. aeruginosa* ATCC 27853	>4

Quality control ranges: *E. coli* ATCC 25922 (0.008–0.03 mg/L) and *P. aeruginosa* ATCC 27853 (1–8 mg/L) [37].

**Table 2:** Steady-state inhibitory kinetics using tebipenem.

Parameter	PenA1	AmpC1
*K*_i app_ (µM)	4.7 ± 0.5	22 ± 2
*k*_2_/*K* (M^−1^s^−1^)	N/D	1.9 ± 0.1 × 10^3^
*k*_off_ (s^−1^)	N/D	3 ± 1 × 10^−4^
*t*_n_ at 15 min	4000	10

Each data point was collected in triplicate, and each experiment was completed in duplicate. N/D, not determinable due to background hydrolysis of tebipenem, see Figure 2.

## Data Availability

Not applicable.

## Supplementary Materials

The following supporting information can be downloaded at: https://www.mdpi.com/article/10.3390/antibiotics11050674/s1, Table S1: Antimicrobial susceptibility testing results for 151 Bcc and *B*. *gladioli* conducted via agar dilution methodology.
