# Supplementary material for: The Class A β-Lactamase Produced by Burkholderia Species Compromises the Potency of Tebipenem against a Panel of Isolates from the United States
Source: Antibiotics (Basel). 2022 May 17;11(5):674. doi: 10.3390/antibiotics11050674 (PMC9137479; doi:10.3390/antibiotics11050674)
Supplement: Supplementary file 1 [file antibiotics-11-00674-s001.zip › antibiotics-1720656-supplementary.pdf]

Table S1. Antimicrobial susceptibility testing results for 151 Bcc and *B. gladioli* conducted via agar dilution methodology.

Strain	TEBI	IPM*	CAZ*	SXT*
Susceptible	0	24	94	92
Intermediate	0	14	14	N/A
Resistant	151	113	43	54
B. ambifaria AU11161	>4	16	128	16/304
B. ambifaria AU19862	4	2	2	0.5/9.5
B. ambifaria AU20319	>4	4	64	1/19
B. ambifaria AU5203	>4	4	4	1/19
B. arboris AU14372	>4	32	8	1/19
B. arboris AU24192	>4	8	4	1/19
B. cenocepacia AU0133	>4	32	4	2/38
B. cenocepacia AU0583	4	16	4	8/152
B. cenocepacia AU0756	>4	128	128	8/152
B. cenocepacia AU10321	>4	32	32	8/152
B. cenocepacia AU11339	4	4	4	1/19
B. cenocepacia AU12265	>4	16	4	2/38
B. cenocepacia AU12571	>4	8	2	0.5/9.5
B. cenocepacia AU12659	>4	4	8	0.5/9.5
B. cenocepacia AU13262	>4	8	16	1/19
B. cenocepacia AU14093	>4	64	128	8/152
B. cenocepacia AU14109	>4	32	32	16/304
B. cenocepacia AU14381	>4	16	8	16/304
B. cenocepacia AU16448	>4	16	4	4/76
B. cenocepacia AU17796	>4	32	4	0.5/9.5
B. cenocepacia AU19276	4	2	8	1/19

B. cenocepacia AU19530	>4	128	16	4/76
B. cenocepacia AU19684	>4	8	32	16/304
B. cenocepacia AU20146	4	8	4	1/19
B. cenocepacia AU20545	4	4	4	1/19
B. cenocepacia AU20902	>4	16	4	0.5/9.5
B. cenocepacia AU20998	>4	16	128	8/152
B. cenocepacia AU21083	>4	8	4	2/38
B. cenocepacia AU22509	>4	16	4	4/76
B. cenocepacia AU24362	>4	64	32	4/76
B. cenocepacia AU28111	4	4	4	1/19
B. cenocepacia AU29265	4	4	4	0.5/9.5
B. cenocepacia AU3663	>4	4	16	4/76
B. cenocepacia AU6550	>4	64	4	8/152
B. cenocepacia AU8635	>4	64	16	8/152
B. cenocepacia AU9215	>4	32	64	16/304
B. cenocepacia AU9292	4	16	4	16/304
B. cenocepacia AU9512	>4	8	16	8/152
B. cenocepacia AU9710	4	8	4	1/19
B. cepacia AU0108	4	8	8	2/38
B. cepacia AU0329	4	8	8	16/304
B. cepacia AU11420	>4	16	16	4/76
B. cepacia AU13163	>4	8	8	0.5/9.5
B. cepacia AU13354	>4	32	64	1/19
B. cepacia AU15249	4	8	8	2/38
B. cepacia AU1555	>4	16	8	8/152
B. cepacia AU15677	4	2	8	0.5/9.5

B. cepacia AU16396	>4	16	8	2/38
B. cepacia AU17630	>4	8	32	1/19
B. cepacia AU19265	4	4	8	2/38
B. cepacia AU22213	4	4	8	1/19
B. cepacia AU23707	>4	32	8	2/38
B. cepacia AU27816	4	4	4	0.5/9.5
B. cepacia AU29493	>4	8	16	0.5/9.5
B. contaminans AU15669	>4	64	16	2/38
B. contaminans AU17641	>4	16	8	2/38
B. contaminans AU20979	>4	8	128	N/T
B. contaminans AU21811	>4	16	64	1/19
B. contaminans AU22443	>4	8	4	2/38
B. contaminans AU22662	4	8	4	1/19
B. contaminans AU25403	4	8	4	1/19
B. diffusa AU19637	4	2	16	2/38
B. dolosa AU12872	>4	128	128	16/304
B. dolosa AU29021	>4	32	32	1/19
B. dolosa AU29985	>4	32	512	8/152
B. dolosa AU9336	>4	16	8	8/152
B. gladioli AU0032	4	1	8	0.5/9.5
B. gladioli AU1009	2	0.5	8	0.5/9.5
B. gladioli AU16341	>4	2	64	N/T
B. gladioli AU21101	1	0.5	8	0.25/4.75
B. gladioli AU26456	1	0.25	16	N/T
B. gladioli AU27927	1	0.25	8	0.25/4.75
B. gladioli AU28659	4	1	16	1/19

B. gladioli AU29223	1	0.5	8	0.25/4.75
B. gladioli AU29541	>4	1	64	N/T
B. gladioli AU30473	4	1	32	0.5/9.5
B. multivorans AU10047	>4	32	8	0.5/9.5
B. multivorans AU10086	>4	64	8	16/304
B. multivorans AU10398	>4	4	512	4/76
B. multivorans AU10897	>4	128	4	8/152
B. multivorans AU11204	4	16	2	1/19
B. multivorans AU11233	>4	64	32	16/304
B. multivorans AU11358	>4	64	2	1/19
B. multivorans AU11772	>4	16	>512	4/76
B. multivorans AU12481	4	16	4	1/19
B. multivorans AU13919	4	32	2	1/19
B. multivorans AU14328	4	16	2	1/19
B. multivorans AU14364	4	1	256	8/152
B. multivorans AU14371	4	64	2	0.5/9.5
B. multivorans AU14786	>4	64	32	4/76
B. multivorans AU15814	>4	64	256	16/304
B. multivorans AU15954	4	32	2	1/19
B. multivorans AU16734	>4	64	2	2/38
B. multivorans AU17135	4	32	2	0.5/9.5
B. multivorans AU17534	>4	64	64	2/38
B. multivorans AU17545	2	8	8	4/76
B. multivorans AU18096	>4	32	2	0.5/9.5
B. multivorans AU19518	>4	32	4	0.5/9.5
B. multivorans AU19564	>4	32	2	2/38

B. multivorans AU19659	>4	64	32	1/19
B. multivorans AU19729	>4	32	8	>16/304
B. multivorans AU20929	4	32	2	2/38
B. multivorans AU21015	>4	64	128	16/304
B. multivorans AU21596	>4	128	64	16/304
B. multivorans AU21747	4	64	2	2/38
B. multivorans AU22436	>4	64	2	0.25/4.75
B. multivorans AU22892	4	64	2	1/19
B. multivorans AU23365	>4	32	32	4/76
B. multivorans AU23668	4	16	1	0.5/9.5
B. multivorans AU23690	>4	16	4	4/76
B. multivorans AU23919	4	4	512	8/152
B. multivorans AU23995	>4	32	8	2/38
B. multivorans AU24277	>4	32	64	4/76
B. multivorans AU25057	>4	32	8	4/76
B. multivorans AU25543	4	2	2	0.25/4.75
B. multivorans AU26250	4	32	2	0.5/9.5
B. multivorans AU26526	>4	32	16	2/38
B. multivorans AU27706	2	0.25	2	1/19
B. multivorans AU28069	4	16	2	1/19
B. multivorans AU28442	>4	64	>128	N/T
B. multivorans AU29198	>4	64	16	4/76
B. multivorans AU30050	4	32	1	1/19
B. multivorans AU30438	4	32	4	0.5/9.5
B. multivorans AU30441	4	32	2	0.5/9.5
B. multivorans AU30760	4	16	2	0.5/9.5

B. multivorans AU4057	>4	64	2	0.5/9.5
B. pseudomultivorans AU19682	>4	8	32	2/38
B. pyrrocinia AU1114	4	16	8	16/304
B. pyrrocinia AU4348	4	32	16	8/152
B. seminalis AU14842	>4	4	4	1/19
B. stabilis AU10235	>4	16	256	4/76
B. stabilis AU9035	>4	16	32	16/304
B. ubonensis AU7314	>4	32	512	2/38
B. veitnamiensis AU10214	4	0.25	8	0.5/9.5
B. vietnamiensis AU19457	>4	16	8	8/152
B. vietnamiensis AU21549	2	0.25	2	1/19
B. vietnamiensis AU26096	>4	16	8	16/304
B. vietnamiensis AU28056	>4	0.5	4	0.25/4.75
B. vietnamiensis AU28891	>4	0.5	4	16/304
B. vietnamiensis AU30387	2	0.125	1	0.5/9.5
B. vietnamiensis AU3578	>4	0.5	64	4/76
B. vietnamiensis AU3997	>4	0.25	2	1/19
B. vietnamiensis AU5003	>4	32	8	16/304
Bcc Indeterminate AU18117	>4	16	32	16/304
Bcc Indeterminate AU12560	>4	8	4	0.5/9.5
Bcc indeterminate AU12848	>4	16	64	1/19
Bcc Indeterminate AU14915	>4	64	32	4/76
Bcc Indeterminate AU14962	>4	16	8	1/19
Bcc Indeterminate AU19821	>4	64	32	2/38
Bcc Indeterminate AU9162	>4	8	8	1/19
Bcc Indeterminate AU19076	4	8	4	2/38

CLSI breakpoints for ceftazidime (CAZ) (S ≤ 8 mg/L, I = 16 mg/L, R ≥ 32 mg/L) and trimethoprim/sulfamethoxazole (SXT) (S ≤ 2/38 mg/L, R ≥ 4/76 mg/L) against Bcc were used (41). CLSI breakpoints for imipenem (IPM) (S ≤ 2 mg/L, I = 4 mg/L, R ≥ 8 mg/L) against *P. aeruginosa* were used, as breakpoints against Bcc are not available (41). Provisional MIC interpretations for tebipenem (TEBI) are as follows: susceptible ≤ 0.12 mg/L, intermediate 0.25 mg/L, and resistant ≥0.5 mg/L.*Data was previously published, SXT was run at a ratio of 1:19 mg/L. N/T = not tested.
